# Intermittent Hypoxic-Hyperoxic Exposures Effects in Patients with Metabolic Syndrome: Correction of Cardiovascular and Metabolic Profile

**DOI:** 10.3390/biomedicines10030566

**Published:** 2022-02-28

**Authors:** Afina Bestavashvili, Oleg Glazachev, Alexander Bestavashvili, Alexander Suvorov, Yong Zhang, Xinliang Zhang, Andrey Rozhkov, Natalia Kuznetsova, Chavdar Pavlov, Dmitriy Glushenkov, Philippe Kopylov

**Affiliations:** 1 Department of Cardiology, Functional and Ultrasound Diagnostics, N.V. Sklifosovsky Institute of Clinical Medicine, I. M. Sechenov First Moscow State Medical University, 119991 Moscow, Russia; tusia.13@bk.ru (N.K.); fjk@inbox.ru (P.K.); 2 Department of Normal Physiology, N.V. Sklifosovsky Institute of Clinical Medicine, I. M. Sechenov First Moscow State Medical University, 119991 Moscow, Russia; glazachev@mail.ru (O.G.); zxl0620@yandex.ru (X.Z.); 3 Department of Therapy, General Practice and Nuclear Medicine, Pirogov Russian National Research Medical University, 117997 Moscow, Russia; alexanderbestavashvili@gmail.com; 4 World-Class Research Center “Digital Biodesign and Personalized Healthcare”, I. M. Sechenov First Moscow State Medical University, 119991 Moscow, Russia; yourmedstat@gmail.com (A.S.); rozhkov_a_n@staff.srchenov.ru (A.R.); 5 The State-Province Key Laboratories of Biomedicine-Pharmaceutics of China, Key Laboratory of Cardiovascular Research, Ministry of Education, Department of Pharmacology, TbalHarbin Medical University, Harbin 150081, China; hmzhangyong@hotmail.com; 6 Department of Therapy of the Institute of Professional Education, I. M. Sechenov First Moscow State Medical University, 119991 Moscow, Russia; chpavlov@mail.ru; 7 Botkinskaya Hospital, 125284 Moscow, Russia; 8 Department of Internal Medicine, Gastroenterology and Hepatology, N.V. Sklifosovsky Institute of Clinical Medicine, I. M. Sechenov First Moscow State Medical University, 119991 Moscow, Russia; dr.glushenkov@gmail.com

**Keywords:** atherosclerosis, metabolism, intermittent hypoxic training, hypoxic-hyperoxic exposures, low-density lipoprotein, inflammation, arterial stiffness, liver fibrosis, liver steatosis

## Abstract

The aim of this study was to evaluate efficacy and applicability of the “intermittent hypoxic-hyperoxic exposures at rest” (IHHE) protocol as an adjuvant method for metabolic syndrome (MS) cardiometabolic components. A prospective, single-center, randomized controlled clinical study was conducted on 65 patients with MS subject to optimal pharmacotherapy, who were randomly allocated to IHHE or control (CON) groups. The IHHE group completed a 3-week, 5 days/week program of IHHE, each treatment session lasting for 45 min. The CON group followed the same protocol, but was breathing room air through a facial mask instead. The data were collected 2 days before, and at day 2 after the 3-week intervention. As the primary endpoints, systolic (SBP) and diastolic (DBP) blood pressure at rest, as well as arterial stiffness and hepatic tissue elasticity parameters, were selected. After the trial, the IHHE group had a significant decrease in SBP and DBP (Cohen’s d = 1.15 and 0.7, *p* < 0.001), which became significantly lower (*p* < 0.001) than in CON. We have failed to detect any pre-post IHHE changes in the arterial stiffness parameters (judging by the Cohen’s d), but after the intervention, cardio-ankle vascular indexes (RCAVI and LCAVI) were significantly lowered in the IHHE group as compared with the CON. The IHHE group demonstrated a medium effect (0.68; 0.69 and 0.71 Cohen’s d) in pre-post decrease of Total Cholesterol (*p* = 0.04), LDL (*p* = 0.03), and Liver Steatosis (*p* = 0.025). In addition, the IHHE group patients demonstrated a statistically significant decrease in pre-post differences (deltas) of RCAVI, LCAVI, all antropometric indices, NTproBNP, Liver Fibrosis, and Steatosis indices, TC, LDL, ALT, and AST in comparison with CON (*p* = 0.001). The pre-post shifts in SBP, DBP, and HR were significantly correlated with the reduction degree in arterial stiffness (ΔRCAVI, ΔLCAVI), liver fibrosis and steatosis severity (ΔLFibr, ΔLS), anthropometric parameters, liver enzymes, and lipid metabolism in the IHHE group only. Our results suggested that IHHE is a safe, well-tolerated intervention which could be an effective adjuvant therapy in treatment and secondary prevention of atherosclerosis, obesity, and other components of MS that improve the arterial stiffness lipid profile and liver functional state in MS patients.

## 1. Introduction

It is well known that Metabolic Syndrome (MS), being a cluster of interrelated pathological conditions such as obesity, hypertension, and lipid profile, and carbohydrate metabolism disorders, is a major risk factor for vascular atherosclerosis, type 2 diabetes mellitus (DM2T), and serious (critical) cardiovascular complications (heart attack, stroke) [1]. According to the World Health Organization, as of 2016, more than 1.9 billion adults (39%) aged ≥18 are overweight, of whom more than 650 million (13%) are obese, and these numbers are annually increasing [2]. In 2019, 460 million people (6.25%) suffered from type 2 diabetes, while approximately 18.6 million deaths were attributed to the leading worldwide cause of death—cardiovascular disease (CVD), which largely results from excess body weight and physical inactivity [3].

Atherosclerosis, as the leading cardiovascular pathology development factor, is a complex pathological process that involves lipid metabolism and mitochondrial dysfunction, as well as chronic inflammation [4]. However, the initial atherosclerotic vascular damage stages, as well as the possibilities of their control, have been insufficiently studied.

One of the most important, slowly progressing conditions associated with both MS and aggravation of lipid and carbohydrate metabolism disorders and DM2T development is the initiation and progression of non-alcoholic fatty liver disease (NAFLD) [5,6,7,8]. Inflammation, oxidative stress and impaired carbohydrate metabolism underline NAFLD pathogenesis. In turn, hepatic steatosis is characterized by atherogenic dyslipidemia: high levels of TG and LDL, and low levels of HDL, increasing obesity and cardiovascular disease risk [9,10]. Since multiple metabolic disorders underlie NAFLD, the term has been introduced to denote it—metabolic-associated fatty liver disease (NAFLD) [11]. The new term stresses the bidirectional relationships and increases awareness in looking for fatty liver disease among patients with MS/DM2T and CVD or separate cardiovascular risk factors, as well as revealing these comorbidities among patients with NAFLD.

A large number of studies aim at developing treatments for cardiometabolic diseases, but the emphasis should be placed on MS as the condition that precedes them, especially since most MS components are known to be reversible [12]. There are solid justifications for multicomponent drug therapy in MS patients, however its side effects are well-reported. Therefore, finding effective non-pharmacological cardiovascular and metabolic risk factors treatment and correction methods is vital [5].

Today, the first-line MS and NAFLD therapy are lifestyle changes—body weight reduction via a healthy diet and/or individualized physical activity regimens [6,7,8,11]. However, low-intensity exercise training, which triggers no significant changes in immunological and physiological reactions and does not lead to the desired effects, remains the only available option for a large number of patients with cardiometabolic pathology accompanied by musculoskeletal disorders [13]. A number of studies have shown that low-intensity exercise combined with hypoxic and/or hyperoxic gas breathing increases metabolic load and oxidative stress in a manner similar to higher-intensity exercise [14,15,16,17,18,19,20]. For instance, it has been shown that moderate-intensity exercise in a hypoxic environment promotes skeletal muscle capillarization, reduces arterial stiffness [17], and leads to significant improvements in lipid metabolism, glucose metabolism, and weight loss in obese patients as compared with controls [20]. However, several summary reviews suggest that training in a hypoxic environment has no proven beneficial effects on weight loss [16], glucose, and lipid homeostasis in patients with T2DT [21]. Therefore, additional well-designed trials are required to establish the optimal protocols of combined physical exercise and hypoxic environment application in different patient categories [19,21,22].

At the same time, these reviews indicate that passive hypoxic exposures (PHE), particularly in the interval mode, may be the method of choice for non-drug correction of cardiometabolic risks in obese patients with DM2T. It has been shown that a course of passive interval hypoxic exposures is effective in increasing insulin sensitivity in patients with prediabetes [23], decreasing blood pressure, and correcting endothelial dysfunction [24]. A pilot uncontrolled study demonstrated that using Interval Hypoxic-Hyperoxic Exposure (IHHE) (a modified protocol for combining short hypoxic and hyperoxic exposures by breathing through a face mask) at rest as a monotherapy without exercise, in treatment of MS patients, led to a significant weight loss due to decrease in fat mass which was accompanied by decrease in Total Cholesterol, Low-Density Lipoproteins, and fasting glucose, as well as blood pressure normalization [25,26]. However, no controlled studies of IHHE effects on metabolic, hemodynamic, and inflammatory status of MS patients have been conducted so far.

The aim of this study was to evaluate the efficacy and applicability of the IHHE protocol as an auxiliary method for MS cardiometabolic components correction, in particular applying to the vascular status, lipid metabolism, and liver functional state indicators.

## 2. Materials and Methods

### 2.1. Study Design

Patient recruitment for the study, all laboratory and instrumental procedures, and the training course were conducted at the Cardiology Clinic, I.M. Sechenov First Moscow State Medical University, Russia. A simple blind prospective randomized (1:1 randomization ratio) controlled trial with participation of 65 (33 male and 32 female) patients with MS aged from 29 to 74 years was carried out. The patients were randomly allocated into two groups: experimental (IHHE) and control. Patients in the IHHE group were subjected to a course of interval hypoxic-hyperoxic exposures (IHHE), 5 procedures per week, for the total of 15 procedures over 3 weeks. Patients in the control (placebo) group were subjected to the same protocol, but instead of intermittent hypoxia-hyperoxia, room air (normoxia, 20.9% O_2_) was supplied in a similar mode.

The main endpoints included changes in hemodynamic parameters: systolic (SBP) and diastolic (DBP) blood pressure, heart rate (HR), changes in arterial stiffness according to CAVI (cardio-ankle vascular index), and liver tissue elasticity and fibrosis stage estimation according to liver elastography. The secondary endpoints included the lipid spectrum (total cholesterol (TC), high-density lipoproteins (HDL) and low-density lipoproteins (LDL), triglycerides, liver transaminases—alanine aminotransferase (ALT) and aspartate aminotransferase (AST), and the inflammation markers (CRP-highly sensitive, and N-terminal prohormone of brain natriuretic peptide level (NT-proBNP).

The study was approved by the I.M. Sechenov First Moscow State Medical University ethical committee (Local Ethical Protocol № 05-19 10.04.2019) and performed according to the ethical standards based on the Helsinki Declaration on Ethical Principles for Medical Research Involving Human Subjects. Written informed consent was obtained from all study participants. The study was registered at ClinicalTrials.gov (NCT04791397, protocol identifier A0519).

### 2.2. Participants and Randomization

Eighty-six patients with metabolic syndrome (MS) aged 29–74 years with a stable clinical condition within the past 3 months were invited to participate in the study. MS was defined according to the National Institute of Health guidelines as the presence of three or more of the following characteristics: waist circumference greater than 89 cm in females and greater than 102 cm in males, blood pressure ≥130/85 mm Hg for SBP and DBP, dyslipidemia (triglyceride level ≥150 mg/dL (1.7 mmol/L); high-density lipoprotein cholesterol (HDL) <40 mg/dL (1.04 mmol/L) in males or <50 mg/dL (1.3 mmol/L)) in females, elevated fasting blood sugar (≥100 mg/dL (5.6 mmol/L)). The exclusion criteria included individual intolerance to hypoxia, liver cirrhosis, Child-Pugh class C, being serologically positive for hepatitis B and C, chronic kidney disease (GFR <30 mL/min/1.73 m^2^), pregnancy, severe respiratory distress, acute cardiovascular conditions, and neuromuscular disorders. The study included 65 patients who were randomly assigned to either the IHHT group (32 patients) or the control group (33 patients). The patients were recruited from March 2019 to March 2020. Due to withdrawn consent, hypoxia intolerance, location inconveniences, and impossibility to perform liver ultrasound analysis, 21 patients were excluded from the study.

**Figure 1:**
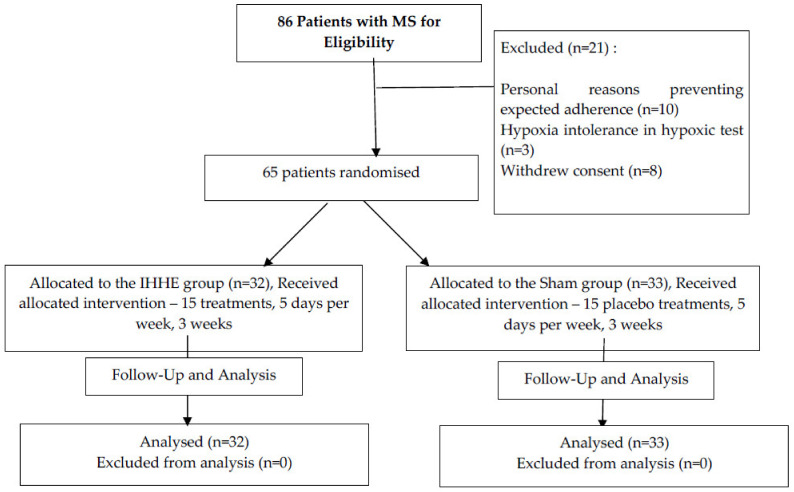
MS—Metabolic Syndrome; IHHE: intermittent hypoxic-hyperoxic exposure.

The process of inclusion, randomization, stratification, IHHE intervention program, and outcome analysis is shown in Figure 1. Baseline anthropometric, clinical characteristics, and medications are presented in Table 1. Groups were matched for sex, age, presence of MS components, and comorbidities. Patients were asked to adhere to their daily food intake, physical activity, prescribed medications, and usual lifestyle for the duration of the study.

#### IHHE Program

Patients included in the study underwent IHHE using the ReOxy respiratory therapy device (AI Mediq S.A., Luxembourg). At the first visit, after all anthropometric and laboratory-instrumental examinations were performed, the patients of both groups were subjected to a hypoxic breath test (HT) for 10 min. The test was performed in a sitting position in an armchair, with a hypoxic gas mixture supplied through a face mask with 11% O_2_ concentration under arterial blood oxygen saturation (SpO_2_) and HR (measured continuously using a KIT Masimo built-in pulse oximeter (measurement error ± 2%) control. Based on the result of the hypoxic test, the device automatically adjusted the individual IHHE procedures mode for each patient. The data on each procedure, starting from the hypoxic test, were stored in the device memory.

Starting from the second day of the study, the patients in the IHHE group were exposed to a gas mixture with a variable oxygen concentration ranging from 10% to 14% (corresponding to 4000–6500 m above sea level) to 35% and nitrogen. Depending on the individual tolerance of hypoxia determined by the day 1 HT, the patients inhaled the hypoxic gas mixture with 11–12% O_2_ content for 4–7 min, followed by 2–4 min inhalation of the hyperoxic gas mixture with 30–35% O_2_ content. The research physician observed every procedure and monitored SpO_2_ and pulse rate, which were recorded and transmitted to the invisible to patients device monitor. When the minimum SpO_2_ value determined by the individual minimum SpO_2_ level was reached, the device automatically switched over to supplying a hyperoxic gas mixture until full pre-hypoxic recovery of the SpO_2_ level occurred (usually within 1–3 min). Then the next hypoxia-hyperoxia exposure cycle was repeated. Each procedure lasted from 40 to 45 min, and included 5–8 hypoxia-hyperoxia cycles. Blood pressure was measured before and after each procedure. The patients in the control group received the procedures according to the same scheme as the patients in the main group, with the same “exposure” time and sessions number, but with a normoxic gas mixture (room air) supplied throughout the session instead.

At the beginning (during the first 1–2 sessions) of the IHHE course, some patients complained of slight transient dizziness and short dyspnea, which did not require any procedure interruption. After the first session, none of the patients refused to continue participation in the study.

### 2.3. Examination Methods

Prior to IHHE/placebo exposure, all patients underwent planned medical examination recording medical history, daily drug therapy regimen, presence of notable cardiovascular disease family history, carbohydrate metabolism disorders, obesity, and other pathologies.

Two days before, and 2–3 days after the last IHHE procedure, both patient groups went through the same examinations and measurements including hemodynamic parameters (blood pressure (systolic—SBP and diastolic—SBP), heart rate (HR), SpO_2_ at rest); anthropometric data (height, body weight, waist and hip circumference), arterial stiffness (pulse wave velocity assessment, CAVI), liver tissue elasticity (stiffness), and fibrosis/steatosis stage evaluation. Venous blood samples were taken to determine serum lipid spectrum, liver enzymes, and individual chronic inflammation markers (CRP-hs and NTpro-BNP).

Heart rate (HR) and blood pressure values in all participants were measured twice after a 5-min rest in a sitting position on both arms using an automatic AND UA-767 tonometer (A&D, Saitama, Japan), and the average values were used as the result values. SpO_2_ levels were recorded using a pulse oximeter (Beurer: PO30, Hollywood, LA, USA). Weight and height were measured with a Seca gmbh&co.kg scale (Hammer Steindamm 3-25, 22089 Hamburg, Germany). Waist circumference was measured in centimeters in between the lower edge of the last palpable rib and the iliac crest apex using a centimeter tape. The hip circumference was measured at the widest part of the thigh in the same way.

### 2.4. Arterial Stiffness Assessment

#### Cardio-Ankle Vascular Index (CAVI)

After a patient rested for at least 5 min in a supine position, noninvasive arterial stiffness measurements were performed using the VaSera VS-1500N (Fukuda Denshi Co., LTD, 39-4 Hongo 3-chome, Bunkyo-ku, Tokyo 113-8483, Japan,) vascular screening system performing analysis of the cardio-ankle vascular index (CAVI) and ankle-brachial index (ABI).

The device measured arterial pressure on four limbs, as well as pulse wave propagation rate from the aortic valve to the arteries of the right and left tibia using plethysmography. Simultaneously, electrocardiograms were recorded by placing electrodes on both arms, and phonocardiography was recorded by placing a high-sensitivity microphone at the second intercostal space on the sternum [27,28].

The study was conducted within 10–15 min on average, while the procedure itself lasted for about 5 min.

The CAVI was calculated automatically by the device as CAVI = a{(2ρ/ΔP) × ln(Ps/Pd)PWV2} + b, where Ps is systolic blood pressures and Pd—diastolic blood pressures, PWV is pulse wave velocity between the heart and ankle, ΔP is Ps–Pd, ρ is blood density, a and b are constants [29].

We used 2 CAVI types:

R-CAVI: CAVI between the aortal valve and the right ankle artery.
L-CAVI: CAVI between the aortal valve and the left ankle artery.

The reference values were taken as follows: CAVI < 8.0—normal, 8.0 ≤ CAVI < 9.0—“borderline” values, >9.0—CAVI exceeded indicating abnormal arterial stiffness.

At the same time, this method measures another indicator of arterial damage—the ABI, which reflects the ratio of systolic pressure in the upper arm to that in the lower leg. The ABI ranges from 1.00 ≤ R/L toABI ≤ 1.40 were considered as normal, ‘borderline’ values of 0.91–0.99 included [30]. The index was calculated automatically on the left and right legs (LABI and RABI, respectively).

### 2.5. Liver Elastometry

The assessment of the functional liver status and liver fibrosis stage and steatosis severity was performed by a noninvasive Vibration-Controlled Transient Elastography (VCTETM) ultrasound method. The measurements were taken using the FibroScan 502 Touch (Echosense, France) device [31,32].

The method is based on liver tissue elasticity measurement (LSMs, E score), which is calculated after 10 measurements of transverse waves spreading through liver tissue with estimation of the mean density value in kPa, scatter of IQR and percentage of valid measurements (SR), and fat/lipid content in liver tissue—the controlled attenuation parameter (CAP score) in dB/meter [33]. Liver tissue elasticity was measured in kPa corresponding to a specific liver fibrosis stage according to METAVIR: F0—absence of fibrosis (elasticity value ≤ 5.8 kPa), F1—initial changes (5.9–7.2 kPa), F2—moderate changes (7.3–9.5 kPa), F3—expressed or advanced fibrosis (9.6–12.5 kPa), and F4—cirrhosis (>12.6 kPa). The specificity and sensitivity of the method at the F3/F4 fibrosis stages approached 100%.

The degree of hepatic steatosis was assessed by the CAP score using the XL sensor, which increases the methods’ diagnostic value for patients with excess subcutaneous fat, and the M sensor [34]. The examination procedure was performed in a supine position with the arm behind the head. The sensor was placed in the VI–VIII intercostal space along the middle axillary line. Liver steatosis was defined as a fat mass comprising ≥5% of wet liver weight, with the following stages of fibrosis defined: S0—no steatosis, S1—minimal steatosis, 5–33%, S2—moderate steatosis, 33–66%, S3—severe steatosis, >66% [31].

### 2.6. Venous Blood Samples Analysis

Samples of venous blood (10 mL) were taken from the forearm median brachial vein and collected into vacuum lithium heparin and EDTA tubes. In order to minimize platelet count, blood was allowed to clot (BD Vacutainer Plus SST), and serum was separated immediately (by centrifugation at 3500 rpm for 15min after sampling, Eppendorf Centrifuge 5702R, Darmstadt Germany), aliquoted, and stored at −80 °C; blood analysis was performed within one month from collection.

To minimize run-to-run variability, samples from each participant were analyzed on a microtiter plate. All laboratory analyses were performed by the University Hospital Blood Analysis Center, a blood biochemistry laboratory certified by the Moscow Health Department.

Levels of the N-terminal prohormone brain natriuretic peptide (NTproBNP, N, mediana = 5.6 pmol/L; Biomedica, Vienna, Austria) were measured using an enzyme immunoassay and a Biochrom Anthos 2020 Jencons Microplate Reader photometer. High-sensitivity C-reactive protein (CRP-hs, milligrams per liter, Beckman Coulter, Inc., 250 S. Kraemer Blvd. Brea, CA 92821 USA, assay range 0.2–160 mg/L) was measured using a Siemens Advia 1800 biochemical analyzer (Siemens Healthcare Diagnostics Inc., Tarrytown, New York, NY, USA) by immunoturbidimetry using latex particles.

Lipid profile and AST/ALT were measured using a Siemens Advia 1800 biochemical analyzer (Siemens Healthcare Diagnostics Inc., Tarrytown, New York, NY, USA) and specific test kits from Siemens Healthcare Diagnostics Inc., Tarrytown, New York, NY, USA for total cholesterol (TC, Ref.: 3.2–5.6 mmol/L), high-density lipoproteins (HDL, reference: >1.56 mmol/L), low-density lipoproteins (LDL, reference: ≤4.2 mmol/L), triglycerides (TG, reference: 0.4–1.7 mmol/L;), ALT (reference: 10–49 units/L), and AST (reference: 0–34 units/L).

### 2.7. Statistical Analysis

All data were analyzed using Python Software Foundation version 3.8 for Windows (Delaware, USA). The results are presented as the mean ± SD. The assumption of normality was verified using Shapiro-Wilk’s W-test. To compare the groups at baseline and post-interventional points, Mann-Whitney’s *U*-test was used for numeric or rank data, while for nominal data chi-squared or Fisher’s exact test was used. To estimate the change magnitude between post-interventional and baseline characteristics, Cohen`s d was calculated. The comparison of pre-post parameters change (deltas) was performed for both groups, since the groups were slightly different at the baseline (Mann-Whitney’s *U*-test). Spearman rank correlation analysis was performed to test the relationships between pre-post deltas for all variables. The values of r ≥ 0.60 were considered a strong correlation; the values of r ≥ 0.40—a moderate correlation; all weak correlations were dismissed. The α-level was set at 0.05 for all statistical analysis.

## 3. Results

Table 2 and Table 3 present descriptive statistics, comparisons, and differences between the IHHE and control group for all variables analyzed.

It should be noted that despite randomized formation, the groups significantly differed in a number of average baseline group values characteristics; therefore, we also analyzed the differences (deltas) of the pre- and post-intervention values in each group.

The baseline values of SBP (*p* = 0.02), DBP (*p* = 0.03), as well as a number of lipid metabolism indices—TCh (*p* = 0.01), LDL (*p* = 0.007), AST (*p* = 0.006) in the IHHE group were significantly higher than those in the control group.

After the IHHE treatment, a decrease in the SBP and DBP (Cohen’s d = 1.15 and 0.7, respectively, *p* < 0.001) was observed, and the resting blood pressure values were significantly lower than in the control group. There was no significant decrease in the arterial stiffness parameters after the IHHE course (as per Cohen’s d), but RCAVI and LCAVI were significantly lower in the IHHE group as compared with the control group. The intergroup comparison of mean indices shifts (pre-post) demonstrated significant differences in the degree of SBP, DBP, HR, and all arterial stiffness parameters decreased, except for the LCAVI.

Postinterventional changes in indicators of metabolism, inflammatory status, and liver condition included significant reductions in cholesterol, LDL, and liver fibrosis and steatosis indices (medium-large Cohen’s d) (Table 3). For other indices, the effects were small or insignificant.

However, after IHHE both ALT (*p* = 0.02) and NTproBNP (*p* = 0.02) values became significantly lower than in controls. The observed pre-intervention indices changes, such as the decreased liver fibrosis and steatosis degrees, and changes in the levels of TC, LDL, ALT, AST, as well as in CRP-hs and NTproBNP cardiac overload indicator were significantly more pronounced (*p* = 0.02 − 0.001) in the IHHE group. No significant changes in hemodynamic and metabolic parameters were detected in the control group during the treatment dynamics.

There were no significant changes in anthropometric indices, as well as intergroup differences after the IHHE course (Table 3). However, statistically significant pre-post changes (delta) in anthropometric indices were detected, including a decrease in the waist/hip circumference (−5 cm/−4 cm, *p* = 0.001) and a decrease in BMI (*p* = 0.001) in the IHHE group as compared with the control group.

**Figure 2:**
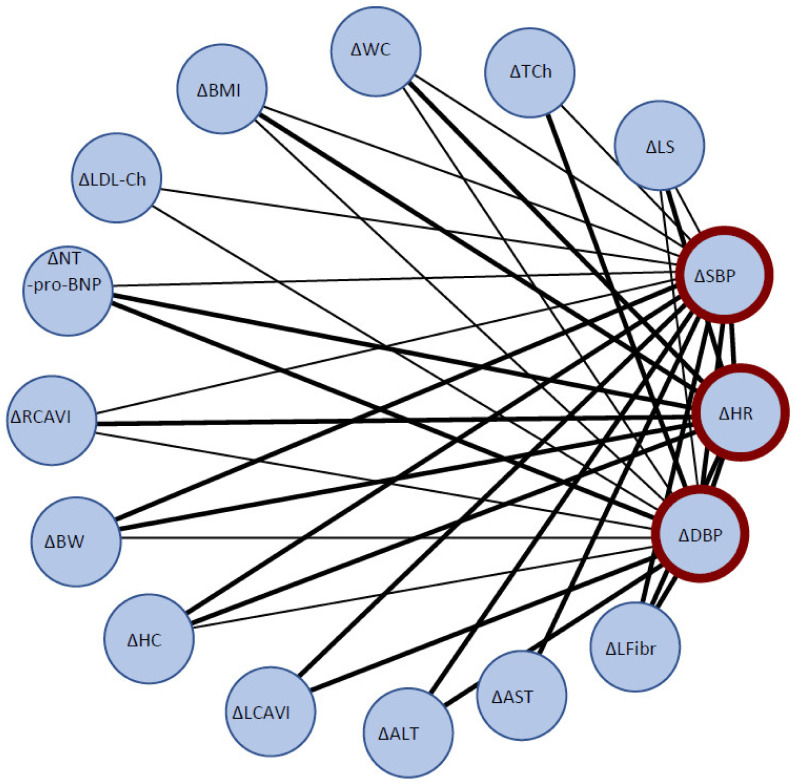
Correlation analysis between pre-post deltas of key hemodynamic parameters (SBP, DBP, and HR) and other variables. Thick lines—strong correlations (r ≥ 0.60), thin lines—moderate correlations (r ≥ 0.40). SBP—systolic blood pressure, DBP—diastolic blood pressure, HR—heart rate, BMI—body mass index, BW—body weight, LS—Liver Steatosis, LFibr—Liver Fibrosis, HC—hip circumference, LDL-Ch—Low-Density Lipoproteins Cholesterol, TCh—Total Cholesterol, WC—waist circumference, RCAVI—right cardio-ankle vascular index, LCAVI—left cardio-ankle vascular index, RABI—right-ankle brachial index, LABI—left ankle-brachial index, ALT—alanineaminotransferase, AST—aspartateaminotransferase, NTproBNP—N-terminal prohormone of brain natriuretic peptide, CRP-hs—High-sensitivity C-reactive protein.

Correlation analysis of the analyzed pre-post-intervention change parameters revealed that in the IHHE group the degree of target indices (SBP, DBP and HR) reduction is directly correlated with the degree of arterial stiffness reduction (ΔRCAVI, ΔLCAVI), liver fibrosis and steatosis severity reduction (ΔLFibr, ΔLS), and the degree of anthropometric parameters, liver enzymes (ΔAST and ΔALT), and lipid metabolism normalization (Figure 2). There were no similar significant correlations in the control group.

## 4. Discussion

Obesity, arterial hypertension, dyslipidemia, and impaired glucose tolerance are the most important risk factors for critical cardiovascular events, and management of these MS components is a substantial problem.

In addition to being traditional CVD risk factors, endothelial dysfunction, atherosclerosis, and vascular lesions can also lead to NAFLD, which is a hepatic manifestation of MS resulting from the associated carbohydrate, lipid, adipokine and proinflammatory cytokine metabolism dysfunction [35]. NAFLD is the most common chronic liver disease, the pathogenesis of which involves inflammation, oxidative stress, and carbohydrate metabolism disorder [9,10]. At the same time, it has been shown that decreased elasticity of hepatic tissue (according to liver stiffness measurement, LSM) and significant fibrosis/steatosis in NAFLD correlate with increased arterial stiffness independently from other common CVD risk factors [36]. Some researchers have proposed that NAFLD can be regarded as an additional component or/and hepatic manifestation of MS [6].

The most adopted treatment methods of MS are lifestyle changes, although other treatments of MS and CVD risk factors have recently emerged and are being studied, including variables protocols of exercise training and intermittent hypoxic exposures [12,17,20,37]. We evaluated the efficacy of passive interval hypoxic-hyperoxic exposures in correction of cardiovascular and hepatic status, as well as metabolic profile in patients with MS under optimal medication. Physical exercises of different modality and intensity performed in a hypoxic gas environment (hypoxic training) are a fairly common nonmedical treatment and rehabilitation approach in patients with MS, obesity, T2DM, and high cardiovascular risk [14,15,18,37,38]. However, the evidence from a number of studies regarding the greater effectiveness of IHT as compared with exercise of the same intensity in normoxia is controversial [17,20,39], and two systematic reviews show that the addition of hypoxia to physical training is not accompanied by more pronounced positive shifts in the lipid and carbohydrate metabolism, anthropometric parameters, and overall health level of obese patients with a high risk of T2DM [19,40]. The authors, however, note the difficulty in comparing different studies results due to use of different IHT protocols in terms of both intensity and frequency of exposure, and the treatment course duration. It should also be noted that patients with MS and obesity are likely to have high orthopedic comorbidity, as well as emotional and motivational limitations in physical activity involvement, which determined our choice of passive IHHE.

In this study, a 3-week IHHE course led to a significant decrease in SBP and DBP, with the blood pressure values in the IHHE group after the intervention being lower than in the control group despite being significantly higher initially. Similar hypotensive effects of an interval hypoxic-normoxic exposures course were noted by N.Lyamina [24], as well as in a recent systematic review which found a decrease in SBP by an average of 13.7 mmHg (*p* < 0.001), and DBP by 7.9 mmHg (*p* = 0.003) in patients with hypertension and coronary artery disease (CAD) after a course of hypoxic exposures [41].

This is corroborated by a N.Muangritdech et al. [42] study showing a decrease in blood pressure and improvement of endothelial function/NO availibility in hypertensive patients both after a 6-week course of interval hypoxic-normoxic exposure at rest, and after a similar IHT course duration.

It has been reported that arterial stiffness is a risk factor for asymptomatic damage of the affected organs [43], and a reliable method of monitoring severity of atherosclerosis and cardiovascular disease, which are the main causes of death among MS patients [44]. The CAVI technique was developed as a more direct measure of stiffness as it provides a ‘pressure-independent’ assessment of the arterial stiffness at the time of measurement, therefore allowing better evaluation of relevant treatment effectiveness [27]. In this study, we did not reveal any significant right and left arterial stiffness indices dynamics in the IHHE group. At the same time, after the IHHE course, RCAVI and LCAVI values were significantly lower, and their pre-post IHHE shifts were also significantly different from controls, which reflects positive dynamics in arterial vascular state and arterial elasticity.

The arterial stiffness reduction is known to decrease heart afterload and normalize left ventricular diastolic function [45]. The RCAVI and LCAVI shifts dynamics observed were accompanied by the reliable differences in NT-pro-BNP shifts (showing decreasing trends in the IHHE group and increasing trends in control), which can reflect reduction of myocardial stretch from volume overload [46]. The values of this marker in the IHHE group after the course were significantly lower than in the control group.

To the best of our knowledge, this is the first study that evaluated the dynamics of liver fibrosis degree and hepatosteatosis severity in patients with MS subjected to a hypoxic conditioning course. We did not reveal a significant fibrosis level reduction in the IHHE group as compared to the baseline data, but a significant steatosis degree reduction was apparent.

In addition, in the post-intervention period, these indices were significantly lower as compared with the control group. Pre-intervention shifts in fibrosis and steatosis indices were significantly more pronounced in MS patients after the IHHE course.

The hepatic tissue is not just a passive target, as its function affects MS pathogenesis and complications, and MS aggravation provokes NAFLD progression. NAFLD is induced by visceral obesity and steatosis which, in combination with insulin resistance, lead to excessive free fatty acids in the liver, fatty dystrophy of hepatocytes, development of oxidative stress with formation of an inflammatory response, and steatohepatitis [47]. To a large extent, it is associated with mitochondrial dysfunction, when microsomal lipid oxidation in the cytochrome system leads to reactive oxygen species generation and increased proinflammatory cytokines production, triggering inflammation and hepatocytes apoptosis due to cytotoxic TNF-alpha1 effects [7,8].

The observed hypoxic conditioning effects on hepatic tissue elasticity and functional status open up new NAFLD treatment prospects. Liver functional status improvement was indicated by liver enzymes (AST and ALT) activity decrease, as well as by a significant reduction of the initially higher TC and LDL values in the IHHE group after a short exposure course. At the same time, positive CRP-hs dynamics suggest chronic inflammation reduction by IHHE.

The study failed to identify any significant IHHE effects on anthropometric indices in MS patients, as there was no significant reduction in body weight and waist and hip circumferences, which is likely to be due to the limited (3 week) course duration. In clinical trials of X.Du [48], C.Lisamore [22], and N. Muangritdech [42] using exercise training combined with IHT in patients with obesity, hypertension and MS, the minimum period required to reduce body weight and fat mass was 6–8 weeks [22,42,48]. Nevertheless, when comparing the shifts in anthropometric indices, some reliable differences were noted, namely their decrease in the IHHE group with almost no changes in control.

It is important to underline that the identified IHHE effects in patients with MS are systemic and interrelated, which is reflected in the results of the correlation analysis of the pre-post treatment shifts in key indicators of improved cardiovascular status, arterial stiffness, functional liver state, and metabolic profile. Such correlations were completely absent in the control group.

The result of correlation analysis of the dynamics of the analyzed parameters (Figure 2) suggests that the degree of normalization of basic hemodynamic indicators (BP and HR shifts during the course of hypoxic conditioning) was significantly associated with the level of reduction in arterial stiffness and NT-pro-BNP (reduction of volume overload), and on the other hand, with reduced values of indicators of hepatic dysfunction, steatosis and fibrosis severity, and normalization of lipid profile of patients (despite a relatively short interventional period of time).

Using data from a number of studies, it can be assumed that a course of passive hypoxic-hyperoxic interventions has a significant systemic effect on all components/phases of NAFLD formation, based on the theory of “three blows”, reduces the degree of steatosis, free fatty acids level, preventing their excessive oxidation with development of oxidative stress and production of pro-inflammatory mediators [49]. In addition, regression of NAFLD has been shown to normalize the production of adipokines (adiponectin, omentin) that suppress vascular inflammation, endothelial adhesion of monocytes, expression of adhesion modules, inhibit the development of atherogenic dyslipidemia [49], and also lead to a decrease in the intima-media thickness [49].

In the present work, we did not analyze the severity of insulin resistance in the examined patients with MS and NAFLD. Nevertheless, a number of studies have shown that this disorder is the leading one in the development of NAFLD and MS with the following formation of cardiovascular pathology [7 Drozdz]. On the other hand, the use of different protocols of adaptation to interval hypoxia in patients with prediabetes, MS, DM2T, and overweight leads to normalization of insulin production in OGTT, HIF-1α dependent enhancement of mRNA expression pyruvate dehydrogenase kinase PDK-1, increased glycolytic efficiency, and HOMA-IR scores [19,20,23,50]. Wherein, in the metabolically compromised group there were significant interactions between improvements in HOMA-IR levels, BMI, arterial stiffness indices, and hypoxia exposure [20].

A number of studies have convincingly demonstrated that IHT or IHHE effectiveness is not limited by the hypoxic stimulus intensity and duration only, as the transitions from hypoxia to reoxygenation, or from normoxia to hyperoxia and vice versa are equally important [50,51]. Cellular hypoxia activates hypoxia-inducible factor-alpha (HIF-1α) signalling cascade triggering induction of multiple protein synthesis genes responsible for nonspecific adaptive response and hormetic reaction [52]. However, the reoxygenation process is accompanied by moderate generation of reactive oxygen species (ROS) which, in physiological concentrations, are signaling molecules which induce a number of transcription factors such as NF-kB, Nrf2, AP-1, and HIF-1α activating expression of numerous protective molecules: antioxidants, BTS, repair enzymes, iron-regulating proteins, etc. It has been demonstrated that intermittent hypoxic stimulation, rather than continuous hypoxic stimulation of the same duration, causes significant hemodynamic and metabolic changes in both healthy elderly people and in patients with various pathologies [24,53].

It is interesting that interval hyperoxia also induces HIF-1α activation followed by a cascade of adaptive shifts, including angiogenesis, stem cell proliferation and migration, glycolytic activity, growth factors production, mitochondrial biogenesis, etc. [51]. It has been suggested that the reversal of moderate hyperoxia back to normoxia is sensed by tissues (cells) as an oxygen deficiency (“relative hypoxia”), which is designated as the “hyperoxic-hypoxic paradox” [51,54].

Recent studies have demonstrated that replacing normoxic intervals with hyperoxic (30–35% O_2_) intervals during interval hypoxic conditioning enhances ROS-induced protective effects without using more pronounced hypoxia levels [55]. It has been shown that IHHE application improves cardiorespiratory fitness in older comorbid cardiac outpatients [56], is better tolerated by prediabetic patients, allows more intensive procedures with increased hypoxic exposure time in comparison to hypoxic-normoxic exposures [49], and has grounds for neuroprotective applications in the elderly [57]. At the same time, IHHE procedures in healthy volunteers are not accompanied by excessive ROS production in comparison with similar procedures where hyperoxia is replaced by normoxia [58].

Possible mitochondrial IHHE adaptation mechanisms should be particularly considered. Recently, the role of mitochondrial dysfunction in the early stages of atherogenic process, including oxidative stress, chronic inflammation, and intracellular lipid accumulation in various cells and tissues (macrophages, smooth myocytes, endotheliocytes, etc.) has been actively discussed, and putative mechanisms of targeted antioxidant and gene therapy of mitochondrial dysfunctions for treatment of dyslipidemia and atherosclerotic vascular disease reviewed [4]. In this regard, the benefits of IHHE may include reduction of oxidative phosphorylation and associated essential activation of glycolysis (UCPs and mitoKATP-mediated modulation of mitochondrial membrane potential and ion homeostasis) during the hypoxic phase, followed by a rapid increase in energy production efficiency in the hyperoxic period, which synergistically improve antioxidant and anti-inflammatory defenses, mitoprotection, and redox signaling [57].

More research is needed to investigate molecular and physiological mechanisms of different hypoxic conditioning regimens and key indicators of IHT/IHHE effectiveness in correction of cardiovascular and metabolic risk factors in MS and CVD, as well as to evaluate effective use of different resting hypoxic conditioning protocols for patients with MS and other relevant nosologies.

### Limitations and Prospects

Despite randomization, the comparison groups in our study significantly differed on a number of indicators assessed. A larger study with the same baseline MS parameters should be conducted to obtain more reliable results. The optimal drug therapy regimen was assessed according to patient reports without checking the actual drug concentrations in patients’ blood, which does not rule out the possibility of increased pharmacotherapy adherence in the experimental group patients. This was a single-centered trial using a limited number of patients, which does not allow firm conclusions in regard to rare but important safety events.

We could not accurately follow the patients’ lifestyle changes, which does not exclude altered adherence to healthy habits.

Patients with severe peripheral artery disease, arrhythmias, asthma, aortic stenosis, and aortic valve insufficiency were not included in the study, due to the poor quality of key indices recording. Patients with phlebothrombosis and severe skin diseases were also not included in the study, due to contraindications for measurements with VaSera VS-1500N.

In addition, the lack of long-term monitoring to evaluate the persistence of the observed effects is a definite limitation of this study.

There may be practical barriers to IHHE use due to the need for special equipment and well-trained staff. To assure patient safety, before IHHE commencement patients should undergo an acute hypoxic test during which electrocardiogram, SpO_2_, and HR are monitored, as was done at the first hypoxic test day of this study.

## 5. Conclusions

In conclusion, we found that using hypoxic conditioning at rest in the intermittent hypoxic-hyperoxic mode was beneficial for correction of cardiovascular and metabolic profile in patients with Metabolic Syndrome. The patients who undertook 15 intermittent hypoxic-hyperoxic exposure treatments had a significant reduction of resting systolic and diastolic blood pressure without any serious side effects or complaints. The observed IHHE group hemodynamic indices improvements were accompanied by remarkably lower arterial stiffness, liver fibrosis and steatosis, and total/LDL-cholesterol indices as compared with the control group. Significant statistical correlations between the degree of pre-post IHHE blood pressure decrease and changes in patients metabolic profile, arterial stiffness, and liver function indicators confirmed the systemic character of adaptational IHHE course effects.

The outcome of this study suggests that controlled hypoxic-hyperoxic treatment is safe, well-tolerated by patients, and could be an effective auxiliary strategy for treatment and secondary prevention of atherosclerosis, obesity, and other metabolic syndrome components by improving patients’ haemoynamics and lipid profile, reducing arterial stiffness, and positively affecting liver function.

## Figures and Tables

**Table 1:** Anthropometric and clinical and characteristics of the patients. The data are expressed as mean ± SD or frequencies (%).

Variables	IHHE Group (*n* = 32)	Sham Group (*n* = 33)	*p*-Value
Sex, male	14 (43.7%)	19 (57.5%)	NS
Age, years	56.9 ± 11.7	59.8 ± 10.3	NS
Smoking	10 (31.2%)	11 (33.3%)	NS
Hypertension	32 (100%)	33 (100%)	NS
Diabetes Mellitus, type 2	6 (18.8%)	10 (30.3%)	*p* = 0.01
Obesity (BMI > 30)	24 (75.0%)	27 (81.8%)	NS
Regular Medication: Aspirin	4 (12%)	8 (24.4%)	NS
ACE inhibitors	16 (50%)	13 (39%)	NS
AT II inhibitors	14 (43.7%)	16 (48.4%)	NS
Calcium channel blockers	12 (37.5%)	12 (36.3%)	NS
Beta-blokers	14 (43.7%)	13 (39.9%)	NS
Diuretics	14 (43.7%)	14 (42.2%)	NS
Statins	14 (43.7%)	17 (51.6%)	NS
Metformine	14 (43.7%)	10 (30%)	NS
Sulfonylureas	6 (18.7%)	4 (12.1%)	NS
Insulin	2 (6.25%)	5 (15%)	NS

Note. IHHE = intermittent hypoxic-hyperoxic training. BMI = body mass index. NS = non-significant differences between groups.

**Table 2:** Pre- and post-intervention data for hemodynamic parameters with the main analysis of covariance results and deltas.

Variables	Groups	Pre-Test	Post-Test	Cohen’s d (95% CI)	*p*-Value	Pre-Post Deltas, Δ	*p* Value (Mann–Whitney U) **
SBP, mm Hg	IHHT	150.1 ± 17.9 * *p* = 0.02	132.5 ± 12.9 * *p* = 0.01	1.15 (0.64, 1.67)	<0.001	−17.6 ± 17.4	<0.001
	Sham	140.7 ± 14.8	141.7 ± 14.0	−0.06 (−0.55, 0.42)	0.979	1.0 ± 9.5	
DBP, mm Hg	IHHT	93.6 ± 10.7 * *p* = 0.03	85.2 ± 10.6 * *p* = 0.02	0.76 (0.26, 1.26)	<0.001	−8.4 ± 8.3	<0.001
	Sham	88.7 ± 9.1	90.1 ± 12.8	−0.12 (−0.61, 0.36)	0.830	1.4 ± 9.7	
HR, bpm	IHHE	71.6 ± 14.1	65.6 ± 12.9	0.46 (−0.03, 0.96)	0.255	−6.1 ± 7.5	<0.001
	Sham	68.6 ± 12.9	68.7 ± 11.7	−0.01 (−0.49, 0.48)	0.999	0.1 ± 10.3	
RCAVI	IHHT	7.9 ± 1.4	7.4 ± 1.3 * *p* = 0.02	0.34 (−0.15, 0.84)	0.516	−0.5 ± 0.3	<0.001
	Sham	8.0 ± 1.5	8.2 ± 1.4	−0.15 (−0.63, 0.34)	0.932	0.2 ± 0.6	
LCAVI	IHHT	7.8 ± 1.4	7.3 ± 1.3 * *p* = 0.02	0.35 (−0.15, 0.85)	0.503	−0.5 ± 0.4	<0.001
	Sham	8.0 ± 1.5	8.2 ± 1.3	−0.12 (−0.6, 0.37)	0.964	0.2 ± 0.6	
RABI	IHHT	1.11 ± 0.08	1.1 ± 0.03	0.19 (−0.30, 0.69)	0.998	−0.01 ± 0.07	0.023
	Sham	1.1 ± 0.1	1.1 ± 0.1	0.04 (−0.65, 0.33)	0.865	−0.0 ± 0.07	
LABI	IHHT	1.13 ± 0.07	1.1 ± 0.06	−0.11 (−0.60, 0.37)	0.961	−0.03 ± 0.08	0.090
	Sham	1.12 ± 0.1	1.12 ± 0.07	0.33 (−0.16, 0.83)	0.547	0.01 ± 0.08	

Values are expressed as mean ± SD. * significant difference between the groups at the same study time (Mann–Whitney U), ** significant difference in pre-post changes (deltas) between the groups.

**Table 3:** Pre- and post-intervention data for metabolic and liver function parameters with the main analysis of covariance results and deltas.

Variables	Groups	Pre-Test	Post-Test	Cohen’s d (95% CI)	*p*-Value	Pre-Post Deltas, Δ	*p* Value (Mann–Whitney U) **
Weight, kg	IHHT	94.6 ± 26.2	95.7 ± 20.7	−0.04 (−0.52, 0.44)	0.997	−1.0 ± 21.0	<0.001
	Sham	99.9 ± 16.3	100.7 ± 16.5	0.11 (−0.38, 0.60)	0.971	0.7 ± 1.7	
BMI	IHHT	34.2 ± 5.2	33.3 ± 5.2	−0.05 (−0.54, 0.43)	0.996	−0.9 ± 0.5	<0.001
	Sham	33.6 ± 4.2	33.8 ± 4.3	0.19 (−0.30, 0.68)	0.865	0.3 ± 0.6	
Waist Circumference, cm	IHHT	116.2 ± 11.2 * *p* = 0.05	111.0 ± 11.6	−0.06 (−0.54, 0.42)	0.994	−5.2 ± 2.4	<0.001
	Sham	113.1 ± 10.6	113.8 ± 10.9	0.46 (−0.03, 0.96)	0.248	0.7 ± 1.8	
Hip Circumference, cm	IHHT	114.1 ± 9.4	110.3 ± 9.4	−0.03 (−0.51, 0.46)	0.999	−3.8 ± 1.7	<0.001
	Sham	112.9 ± 10.6	113.2 ± 11.0	0.37 (−0.13, 0.86)	0.459	0.3 ± 1.0	
Liver Fibrosis, (LSM), kPa	IHHT	12.1 ± 13.6	5.7 ± 2.7 * *p* = 0.05	0.43 (−0.07, 0.93)	0.315	−6.4 ± 12.6	<0.001
	Sham	12.9 ± 18.2	13.1 ± 18.0	−0.02 (−0.50, 0.47)	0.999	0.2 ± 1.0	
Liver Steatosis, stage	IHHT	1.5 ± 1.5	0.5 ± 1.0 * *p* = 0.01	0.71 (0.21, 1.22)	0.025	−1.0 ± 1.0	<0.001
	Sham	1.15 ± 1.5	1.18 ± 1.5	−0.02 (−0.51, 0.46)	0.999	0.0 ± 0.3	
Total Cholesterol, Mmol/L	IHHT	6.0 ± 1.3 * *p* = 0.01	5.1 ± 1.3	0.68 (0.18, 1.18)	0.036	−0.8 ± 0.8	<0.001
	Sham	4.8 ± 1.2	5.1 ± 1.1	−0.24 (−0.72, 0.25)	0.772	0.3 ± 1.0	
LDL-Cholesterol, Mmol/L	IHHT	3.8 ± 1.2 * *p* = 0.007	3.0 ± 1.2	0.69 (0.18, 1.19)	0.034	−0.8 ± 0.7	<0.001
	Sham	2.7 ± 1.1	3.0 ± 1.0	−0.24 (−0.73, 0.25)	0.764	0.3 ± 0.8	
ALT, u/L	IHHT	37.3 ± 26.1	29.0 ± 15.3 * *p* = 0.02	0.389 (−0.11, 0.89)	0.407	−8.3 ± 14.6	<0.001
	Sham	30.8 ± 19.7	36.2 ± 21.5	−0.25 (−0.74, 0.24)	0.738	5.4 ± 9.2	
AST, u/L	IHHT	31.4 ± 19.0 * *p* = 0.006	26.9 ± 10.9	0.288 (−0.21, 0.78)	0.657	−4.5 ± 12.1	<0.001
	Sham	24.9 ± 14.4	28.1 ± 16.0	−0.20 (−0.69, 0.28)	0.841	3.2 ± 6.3	
CRP-hs, mg/L	IHHT	3.608 ± 3.441	2.237 ± 1.527	0.41 (−0.08, 0.91)	0.157	−1.371 ± 3.534	0.012
	Sham	3.51 ± 4.06	3.38 ± 3.49	0.04 (−0.45, 0.528)	0.998	−0.135 ± 1.803	
NTproBNP, pmol/L	IHHT	27.8 ± 44.9	20.36 ± 33.9 * *p* = 0.02	0.15 (−0.35, 0.64)	0.638	−7.1 ± 13.6	<0.001
	Sham	26.16 ± 45.74	35.0 ± 61.9	−0.18 (−0.67, 0.306)	0.881	9.0 ± 18.0	

Values are expressed as mean ± SD. * significant difference between the groups at the same study time (Mann–Whitney U), ** significant difference in pre-post changes (deltas) between the groups.

## Data Availability

Raw data supporting the conclusions of this publication will be made available by the authors without undue reservations.
